# Longitudinal and cross sectional assessments of health utility in adults with HIV/AIDS: a systematic review and meta-analysis

**DOI:** 10.1186/s12913-014-0640-z

**Published:** 2015-01-22

**Authors:** Bach Xuan Tran, Long Hoang Nguyen, Arto Ohinmaa, Rachel Marie Maher, Vuong Minh Nong, Carl A Latkin

**Affiliations:** Johns Hopkins Bloomberg School of Public Health, Baltimore, MD USA; Institute for Preventive Medicine and Public Health, Hanoi Medical University, Hanoi, Vietnam; School of Medicine and Pharmacy, Vietnam National University, Hanoi, Vietnam; School of Public Health, University of Alberta, Edmonton, Alberta Canada

**Keywords:** Quality of life, Utility, HIV, Longitudinal meta-analysis, Systematic review

## Abstract

**Background:**

Utility estimates are important health outcomes for economic evaluation of care and treatment interventions for patients with HIV/AIDS. We conducted a systematic review and meta-analysis of utility measurements to examine the performance of preference-based instruments, estimate health utility of patients with HIV/AIDS by disease stages, and investigate changes in their health utility over the course of antiretroviral treatment.

**Methods:**

We searched PubMed/Medline, Cochrane Database of Systematic Review, NHS Economic Evaluation Database and Web of Science for English-language peer-reviewed papers published during 2000–2013. We selected 49 studies that used 3 direct and 6 indirect preference based instruments to make a total of 218 utility measurements. Random effect models with robust estimation of standard errors and multivariate fractional polynomial regression were used to obtain the pooled estimates of utility and model their trends.

**Results:**

Reliability of direct-preference measures tended to be lower than other types of measures. Utility elicited by two of the indirect preference measures - SF-6D (0.171) and EQ-5D (0.114), and that of Time-Trade off (TTO) (0.151) was significantly different than utility elicited by Standard Gamble (SG). Compared to asymptomatic HIV patients, symptomatic and AIDS patients reported a decrement of 0.025 (p&#×2009;=&#×2009;0.40) and 0.176 (p&#×2009;=&#×2009;0.001) in utility scores, adjusting for method of assessment. In longitudinal studies, the pooled health utility of HIV/AIDS patients significantly decreased in the first 3 months of treatment, and rapidly increased afterwards. Magnitude of change varied depending on the method of assessment and length of antiretroviral treatment.

**Conclusion:**

The study provides an accumulation of evidence on measurement properties of health utility estimates that can help inform the selection of instruments for future studies. The pooled estimates of health utilities and their trends are useful in economic evaluation and policy modelling of HIV/AIDS treatment strategies.

**Electronic supplementary material:**

The online version of this article (doi:10.1186/s12913-014-0640-z) contains supplementary material, which is available to authorized users.

## Background

The rapid scale-up of antiretroviral treatment (ART) services globally has brought about substantial progress in care and treatment for HIV+ patients, transforming HIV/AIDS from a terminal illness into a chronic illness [1,2]. With ART, patients can be socially and economically productive, and thus have not only a longer life, but also a better quality of life. Given this change in the nature of the disease, monitoring of HIV treatment must consider not only the prevention of death but also the maximization of the patients’ quality of life. Traditionally, monitoring HIV treatment has considered medical outcomes and objective indicators, such as treatment retention, viral load, CD4 levels and death [3]. However, health-related quality of life (HRQL) has become a crucial complementary indicator for monitoring health services and patient-related outcomes, and evaluating effectiveness of health interventions in HIV+ populations. Since HIV disease has social and structural components, it is important to have measures that can capture this complexity.

While in general quality of life is an abstract concept that is difficult to quantify, health-related quality of life (HRQL) is a concept that researchers and clinicians have used to assess a patients’ ability to function in their daily life and their perceived well-being [4]. Many different tools have been developed for the measurement of HRQL, and although they vary widely, it is common that HRQL is multi-dimensional that captures all the relevant areas of a patient’s life, including physical health, mental health and functioning, social interaction and role functioning, and general well-being [5]. HRQL can be assessed using generic or condition specific measures. Generic measures are those that are applicable to the general population and large variety of diseases, while condition-specific measures are concerned with issues and symptoms involved with a specific disease. Generic measures can typically be categorized as health status profiles, in which each domain of a patients’ HRQL is scored separately, or as preference-based HRQL (utility) measures, in which patients’ individual scores are preference weighted to achieve an aggregate single score [6]. In health assessment, utility is defined as “a cardinal measure of the preference for, or desirability of, a specific level of health status or specific health outcome”. Utility is defined as a function of health status and the consumption of goods, services, and leisure over a specified period of time [7]. Utility measures are classified by two major approaches: the direct and indirect preference. Direct preference-based measures ask the patients about the value they attach to their current subjective health states. Meanwhile, indirect preference-based approaches use preferences from other samples, usually from general population, to generate preference index scores for hypothetical health states from a HRQOL instrument [8].

Various generic and disease-specific HRQL measures have been applied in HIV populations [5,9-11], most of which, however, were developed before the advent of ART. As a result, the breadth of these measures might include aspects of HRQL which are now less relevant, while lack increasingly important issues in HIV care and treatment [11]. For example, HIV patients may have concerns with sexual functioning, stigma, or body image, and their HRQL may be negatively affected by some of the side-effects of antiretroviral medication [5,9]. In addition, some important methodological considerations of HRQL measures have emerged, such as their sensitivity or responsiveness, and the appropriateness of repeated use in HIV populations [12]. Since many clinical interventions for HIV patients result in small, but significant changes, it is important that HRQL measures used in HIV/AIDS populations are sensitive to such treatment changes [9]. Additionally, since HIV is a progressive and episodic disease, with different symptoms appearing at different times, any HRQL tool must also be responsive to patients’ disease states over time. Finally, the ability of a tool to capture changes in HRQL over time is complicated by the fact that patients often get acclimated to their own disease state, and thus rate their current health as higher although there has not been any change in clinical health status [3].

One of the most important uses of HRQL assessments in the sphere of HIV/AIDS is in decision making about the effectiveness and cost-effectiveness of treatments and interventions [13]. Generic, preference-based measures provide a single summary score of HRQL outcomes, an integral part of the quality-adjusted life-year (QALY) estimation, a measure which has been widely used in cost-effectiveness analyses of health interventions [8,14]. Although utility approaches have been increasingly applied in HIV interventions [15-18], measurements indicate a wide range of scores and use a wide range of methods [15,16]. Therefore, pooled estimates of utility measures both aggregate this data and maximize their external validity, making them more relevant and useful for policy makers, and researchers making economic evaluations of HIV interventions [19].

Previous reviews have compared various instruments in HIV studies [9,11,12,20], however, they did not sufficiently identify the applications of preference-based HRQL measures [9,11,21], nor examine the longitudinal changes in HRQL over time of these measures [16]. We hypothesized that the choices of indirect- and direct- preference based HRQL measures might yield significantly different utility scores, and that utility of patients deteriorated as the disease progressed, and could be improved given antiretroviral treatment. The objectives of this study were to systematically review utility measures applied in HIV studies, estimate health utility of HIV/AIDS patients by disease stages, and investigate changes in their health utility over the course of antiretroviral treatment.

## Methods

### Eligibility criteria

This review followed the PRISMA (Preferred Reporting Items for Systematic Reviews and Meta-Analyses) guidelines when selecting studies for inclusion [22]. Studies were included if 1) they were written in English in the period of 2000 up to February 2014 and accessed following our search strategy; 2) they were longitudinal or cross-sectional design studies, employing preference-based instruments of health utility and reporting the composite score of health utility, 3) their sample included adult participants (≥18 years old) and 4) their full-text articles were available. To minimize the file-drawer effect, we contacted principle investigators of studies on health utility and HIV/AIDS identified but no paper or report published. In addition, we specifically searched for current well-known utility measures that have been applied to HIV populations, including indirect utility measures such as: EuroQol (EQ-5D-3L and EQ-5D-5L), Health utility index (HUI), Quality of Wellbeing (QWB), Short form-6D (SF-6D), 15D; and direct utility measures such as: Standard Gamble (SG), Time trade-off (TTO) and Visual Analogue Scale (VAS). Studies were excluded if they 1) were letters, opinion pieces, editorials, ecological studies, abstracts, and conference proceedings and full reports were not available; 2) were systematic review or meta-analysis studies; 3) used non-utility measures and 4) reported health utility from proxies (e.g. doctors or caregivers). Due to accessibility, we limited our search strategies only for English-language papers. Since a previous study by Tengs and Lin did synthesize utility estimates among HIV/AIDS patients till 2000, we restricted our search for those studies published after 2000 [16].

### Information sources and search strategy

Two separate search strategies were performed, including: 1) searching with a combination of free text keywords and 2) searching for the application of well-known utility measures in HIV/AIDS field. The search process was conducted from 15th February, 2014 to 8th March, 2014 (date of last search). Four databases were used for the search process, including PubMed/Medline, Cochrane Database of Systematic Review, NHS Economic Evaluation Database and Web of Science. The search terms used are listed in Table 1. The search strategy was modified for each database by experienced experts and librarians. Finally, the bibliographies of selected papers were reviewed and the authors of unpublished papers were contacted to identify all of potential relevant studies.

**Table 1:** **Keywords used for search process**

**General term**	**Health utility term**	
**MeSH**	**MeSH**	
HIV infection	Quality of life	
Antiretroviral therapy, highly active	Quality-adjusted life year	
**Title/Abstract**	**Title/Abstract**	
Human immunodeficiency virus	Health-related quality of life	SF-6D
HIV	HRQoL	Health utility index
Acquired immunodeficiency syndrome	Health utility	HUI
Antiretroviral therapy	Utility scores	HUI2
	Utility assessment	HUI3
	Utility measure	15D
	Preference based	Quality of well-being
	Utility based	QWB
	Preference elicitation	Standard gamble
	Cost utility analysis	SG
	QALY	Time trade-off
	Quality adjusted life years	TTO
	Euroqol	Visual analog scale
	Eq-5d	Visual analogue scale
	Eq5d	VAS/RS
**Time**	2000-2014	
**Language**	English	

### Study selection

After the search was completed, all duplicated studies were removed. Next, titles and abstracts of all remaining studies were screened by the research team to ensure that they matched the selection criteria. All papers whose title and abstract revealed that it did not match the selection criteria were excluded. Several further studies were excluded if their full-text articles revealed that they did not measure utility or duplicated data.

### Data items and data collection

Using a data extraction form, three independent reviewers extracted specified data from the final selected studies. These reviewers compared their extraction results, discussing and resolving any disagreements prior to producing the final data file for the statistical analysis Reliability of the data extraction among the three independent reviewers was 90%.

Data collected included information about study setting, study design, sample size, utility measure used, mean or median utility scores, standard deviations, methods of assessment, length of follow-up, and clinical and demographic characteristics of respondents. We collected some additional information about the measures used, including data about validity, reliability and responsiveness of each measure (if available).

To define the health utility of each subject based on clinical characteristics, we divided subjects into 3 disease stage categories: asymptomatic, symptomatic and AIDS. However, when we coded disease stage, we found that HIV/AIDS status was reported in numerous ways. For example, some of articles simply reported their cohorts into 3 groups (asymptomatic HIV infection, symptomatic HIV infection, and AIDS) [23], while some authors reported CD4 cell count or the presence of HIV/AIDS-defining illnesses. In the latter case, we used all available data to identify the health state based on the current Centre for Disease Control and Prevention (CDC) guidelines [23]. If authors described subjects without indicating data about HIV/AIDS stages or CD4 counts, the HIV/AIDS status was classified as “combined stages”. If two articles described overlapping research findings from the same dataset, we removed the article that reported less methodological information.

### Data analysis

We used two approaches in analyzing the data. The first one aimed to obtain the pooled estimates of utility and examine the influences of study characteristics on these estimates [24]. We consider every assessment using a specific tool in both cross-sectional and longitudinal studies as a single measurement, making a dataset of 218 observations. Since most studies actually applied several HRQL measures, these studies were considered as clusters in the model, in which each within-study measurement was seen as a nested observation [25]. Therefore, we conducted meta-regression analysis, using a random effect model with robust estimation of standard error. If the standard deviation of the estimated utility was missing, we calculated it using standard error or 95% confident interval of the estimated utility. In the first model, comparison of individual measure was conducted. Second, we fit separate models for each of the subgroups of interest and adjusted for type of HRQL measure. Finally, we included all study characteristics in a multivariate model. The second approach was applied for longitudinal measurements (n = 99) to estimate the changes in health utility of patients during ART. Traditionally, regression models often provide a linear dose–response relationship that might not truly reflect the variability of health outcomes given different time on ART. To better describe the association between utility scores and duration on ART, we applied multiple fractional polynomials models which are Intermediate between polynomials and non-linear curves. We fitted first-order and second-order fractional polynomial regression with powers (−2,-1, −0.5, 0, 0.5, 1, 2, 3) for the “duration on ART” to increase the flexibility in estimating the best-fitting curve to the health utility trajectories. Data were analyzed using STATA 12.0, ‘xtmixed’ and ‘mfp’ syntax. The details of data analysis and extracted data set are provided in Additional files 1 and 2.

### Ethical approval

All data included in this review were previously published and publicly available. We only synthesize and analyzed aggregated data. Therefore, this study did not require ethical approval.

## Results

**Figure 1:**
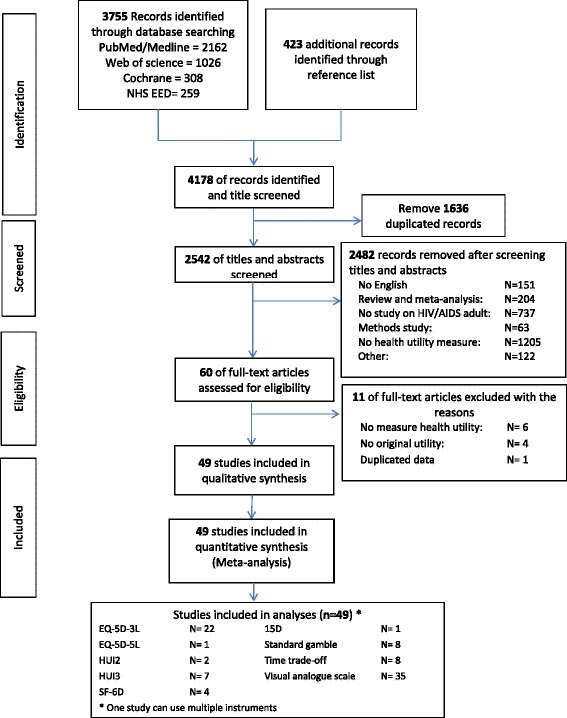
**Flow of study selection.**

Our systematic literature search yielded 49 studies for inclusion in this study (see Figure 1 for flow chart of the search). We selected these studies for their application of nine utility instruments to the field of HIV. These utility measures included 6 indirect and 3 direct preference-based measures (see Table 2 for descriptions of the measures and their psychometric properties). Of the 49 total studies, 14 utilized longitudinal designs, while 37 studies were cross-sectional, generating 218 utility estimates.

**Table 2:** **Overview of selected health utilities measures applied in adults with HIV/AIDS**

**Measures**	**Country of origin**	**Domain**	**No. items**	**Response options**	**No. health states**	**Ranges**	**Endpoints**		**Admin (time)**
							**1 (100)**	**0**	
1. EQ-5D-3L(EuroQol -five dimensions-3 levels)	EuroQoL Group	mobility, self-care, usual activities, pain/discomfort and anxiety/depression	5	3 levels	243	−0.59 to 1.00	full health	death	I, SA* (2 mins)
2. EQ-5D-5L(EuroQol -five dimensions-5 levels)	EuroQoL Group	mobility, self-care, usual activities, pain/discomfort and anxiety/depression	5	5 levels	3125	−0.45 to 1.00	full health	death	I, SA* (2 mins)
3. 15D	Finland	breathing, mental function, speech (communication), vision, mobility, usual activities, vitality, hearing, eating, elimination, sleeping, distress, discomfort and symptoms, sexual activity, and depression	15	5 levels	31 billions	0.00 to 1.00	full health	death	I, SA* (5–10 mins)
4. Health Utility Index Mark 2 (HUI2)	Canada	sensation, mobility, emotion, cognition, self-care, pain and fertility	7	3-5 levels	972,000	−0.02 to 1.00	full health	death	I, SA* (5–10 mins)
5. Health Utility Index Mark 3 (HUI3)	Canada	Vision, hearing, speech, ambulation, dexterity, emotion, cognition, and pain	8	5–6 levels	972,000	−0.36 to 1.00	full health	death	I, SA* (5–10 mins)
6. Short form 6 (SF-6D)	UK	physical functioning; role limitations; social functioning; pain; mental health and vitality	11	4-6 levels	18000	0.00 to 1.00	best health state	worst health state	I, SA* (2 mins)
7. Standard Gamble (SG)	USA	-	-	Continuous	-	0.00 to 1.00	full health	death	I, SA* (2 mins)
8. Time trade-off (TTO)	USA	-	-	Continuous	-	0.00 to 1.00	full health	death	I, SA* (2 mins)
9. Visual analog scale (VAS)	EuroQoL Group	-	-	Continuous	-	0 to 100	full health	worst health	I, SA* (1 mins)

*I: Interview, SA: Self-administered.

Of these 218 utility measures, 8 were of asymptomatic patients, 15 were of symptomatic patients, 56 were from AIDS patients, and 139 were of a combination of patients of different stages (Table 3). VAS accounted for the majority of utility measures (100 times, 45.9%), while HUI2 was only used in 1 measure (0.5%).

**Table 3:** **Characteristics of selected utility measurements**

**Category**	**Characteristic**	**Number of utility measures (n = ** **218)**	
		**Number**	**Percentage**
**Disease stage**	Asymptomatic	8	3.7%
	Symptomatic	15	6.9%
	AIDS	56	25.7%
	Not reported/Combined*	139	63.8%
**Measures**	EQ-5D-3L	53	24.3%
	EQ-5D-5L	3	1.4%
	HUI2	1	0.5%
	HUI3	20	9.2%
	15 D	2	0.9%
	SF-6D	7	3.2%
	SG	15	6.9%
	TTO	17	7.8%
	VAS	100	45.9%
**Settings**	Developed countries	168	77.1%
	Developing countries	50	22.9%
**Study design**	Cross-sectional	119	54.6%
	Longitudinal	99	45.4%

*Data were reported for patients at various disease stage categories.

The majority of utility measures were conducted in developed countries (i.e. USA, UK, Canada, etc.) (with n = 168; 77.1%). 119 utility measures (54.6%) were from cross-sectional studies and 99 (45.4%) were from longitudinal studies.

### Psychometric properties of utility measures in HIV population

Few studies have reported the reliability of these measures. Stavem (2005) [17] determined that the test-retest reliability of EQ-5D, 15D and SF6D was 0.78, 0.90 and 0.94 respectively. Among direct utility measures, Lara (2008) showed a low reliability of 0.41 for SG while it was around 0.71-0.83 for TTO and VAS [16]. Many studies evaluated the validity of utility measures using concurrent and predictive validation. Several studies established convergent validity of EQ-5D, EQ-VAS, HUI3, SG, TTO and VAS by demonstrating their correlation with the subscales of the condition specific MOS-HIV [17,26,27]. In addition, the EQ-5D and HUI3, along with 3 direct preference-based measures, were shown to discriminate subjects by disease severity according to the levels of CD4+ and viral load. Finally, the EQ-5D single index, 15D and SF-6F demonstrated responsiveness relative to a global rating of change [18], while the EQ-VAS and HUI3 demonstrated responsiveness to the development of opportunistic infections, clinical AIDS-defining events, and adverse events [18,26,27] (Table 4).

**Table 4:** **Psychometric properties of selected health utilities measures in HIV population**

**Tools**	**HIV/AIDS population**			**Reliability**		**Validity**				**Longitudinal validity**		**Floor**	**Ceiling**
	**Subjects**	**Country**	**Sample size**	**ICR**	**TTR**	**Construct**	**Criterion (concurrent)**			**Sensitivity**	**Responsiveness**		
						**Convergent**	**Other measures**	**HIV clinical signs**	**Other clinical characteristic**				
EQ-5D-3L*	HIV+ patient [18,26-46]	Developing [37-39,41,46]	16-2261	0.81-0.86 [39]	0.78 [18]	MOS-HIV [27]	SF36 (r = 0.55-0.74) [18]	CD4 count [18,28,38]	HIV stages [29,36]	Improve over time [30]	Decline after diagnosis of AE [26,27,35,40]	0.0 [18,26-28,37,47]	12.4 - 39.7 [18,26-28,32,37,47]
							SF6D (r = 0.74) [18]		GBV-C status [31]				
									ART* status [33]		Decline after health status worse [18]		
							WHOQOL-BREF (r = 0.31-0.60) [37]						
									SAE status [34]		Decline after CD4 and VL decline [32]		
							AQOL (r = 0.539) [41]	Viral load [28]	CD4 count group [32,37]				
		Developed [18,26-36,40,42-45,47]					HUI3 (r = 0.551) [41]				Improve when CD4 improve [38]		
							VAS (r = 0.41-0.80) [18,28,29,39]						
									Viral load group [32]		Decline before and after diagnosis of SAE* [35,40]		
							MOS-HIV (r = 0.40-0.72) [26,28,29,32,47]						
EQ-5D-5L*	HIV+ patients [48]	Developing [48]	1016	0.85 [48]	-	-	VAS (r = 0.73) [48]		HIV stages [48]	-	-		
									CD4 count group [48]				
							Global rating of HRQoL (r = 0.36) [48]						
									Duration of ART [48]				
15D	HIV+ patients [18]	Developed [18]	60	-	0.9 [18]	-	SF36 (r = 0.59-0.80) [18]	CD4 count [18]			Change follow the change of health status [18]	0 [18]	10-12 [18]
							VAS (r = 0.73) [18]	Viral load [18]					
HUI2*	HIV+ patients [34]	Developed [34]	57	-	-	-	-	-		-	-		
HUI3*	HIV+ patient [26,35,40,41,49]	Developing [41]	57-1200	-	-	-	MOS-HIV (r = 0.34-0.70) [26,47]	CD4 count [35,40,49]			Decline after diagnosis of AE* [26]	0-3.2 [26,47]	3.15-5.4 [26,47]
		Developed [26,35,40,47,49]					AQOL (r = 0.543) [41]	Viral load [40]			Decline before and after diagnosis of SAE* [35,40]		
							EQ-5D-3L (r = 0.551) [41]						
SF-6D*	HIV+ patients [4,18,50]	Developed [4,18,50-52]	55-2508	-	0.94 [18]	-	SF36 (r = 0.73-0.79) [18]	-		-	Change follow the change of health status [18]	0 [18]	6-10 [18]
	HIV+												
	women [51]						VAS (r = 0.75) [18]						
	HIV+ IDUs [52]												
SG*	HIV+ patients [4,6,17,26,37,40,53-56]	Developing [17,37,55]	75-450	-	0.41-0.42 [17]	-	TTO (r = 0.21-0.39) [17]		CD4 count groups [26,37,53]	-	Decline after diagnosis of SAE* [40]	7.6-11 [26,37,47]	0.8-22 [26,37,47]
							VAS (r = 0.26-0.34) [17]						
							MOS-HIV (r = 0.14-0.15) [26,47]						
		Developed [4,6,26,40,47,53,54,56]											
							WHOQOL-BREF (r = 0.09-0.34) [37]		HIV stages [4]				
							Global rating of change [17]						
TTO*	HIV+ patients [6,17,26,40,53,54,56,57]	Developing [17]	66-450	-	0.71-0.83 [17]	-	Global rating of change [53]	CD4 count [40]	CD4 count group [26,53]	Improve over time [17]	Decline after diagnosis of SAE* [40]	4.4 [26,47]	18.3 [26,47]
							SG (r = 0.21-0.39) [17]						
		Developed [6,26,40,47,53,54,56,57]					VAS (r = 0.45-0.61) [17]						
							MOS-HIV (r = 0.21-0.29) [26,47]						
VAS*	HIV+ patient [4,6,17,26-30,37,39,40,42,44-46,48,53,54,56,58-71]	Developing [17,37,39,46,58,63,71]	16-2865	-	0.71-0.83 [17]	MOS-HIV [27]	Global rating of change [17]	CD4 count [28,40,61,64]	HIV stages [4,29]	Improve over time [58]	Decline after diagnosis of AE* [26,27] and OI* [27]	0-2 [18,26-28,37,47]	3.3-10.8 [18,26-28,37,47]
							MOS-HIV (r = 0.33-0.72) [26,28,29,47,63]						
							EQ-5D-3L (r = 0.41-0.63) [28,29,39]		CD4 count groups [37,53]				
							EQ-5D-5L (r = 0.73) [48]						
		Developed [4,6,26-30,34,40-42,44,45,47,53,54,56,60,61,64-70]							HIV-RNA groups [64]				
							SG (r = 0.26-0.34) [17]	Viral load [28,64]			Decline before and after diagnosis of SAE* [40,64]		
							TTO (r = 0.45-0.61) [17]						
							WHOQOL-BREF (r = 0.36-0.54) [37]						

*EQ-5D-3L: EuroQol −5 dimensions-3 levels; EQ-5D-5L: EuroQol −5 dimensions-5 levels; HUI2: Health utility index 2; HUI3: health utility index 3; SF-6D: Short form 6-dimensions; SG: standard gamble; TTO: time trade-off; VAS: visual analogue scale; ART: Antiretroviral therapy; VL: viral load; AE: Adverse events; ADE: AIDS defining events; OI: Opportunistic infection; SAE: serious adverse events.

### Utility estimates

Data from the 218 utility measurements of 27,951 subjects were extracted for meta-analysis. The meta-regression results are shown in Table 5, including Model 7 for comparison of individual measure, Model 2-6 for the subgroups of interest and adjusted for type of HRQL measure and Model 1 for all characteristics.

**Table 5:** **Adjusted effect size by duration of follow-up**

			**Model 1**	**Model 2**	**Model 3**	**Model 4**	**Model 5**	**Model 6**	**Model 7**	**Model 8**
	**N**	**%**	**Coefficient (95% CI)**	**Coefficient (95% CI)**	**Coefficient (95% CI)**	**Coefficient (95% CI)**	**Coefficient (95% CI)**	**Coefficient (95% CI)**	**Coefficient (95% CI)**	**Coefficient (95% CI)**
**Const**			**0.784 (0.703; 0.865)**	**0.624 (0.508; 0.740)**	**0.653 (0.562; 0.744)**	**0.639 (0.535; 0.742)**	**0.696 (0.555; 0.838)**	**0.612 (0.454; 0.77)**	**0.616 (0.463; 0.768)**	
**Measures**										
SG	20	8.6								
VAS	81	34.6	0.037 (−0.021; 0.095)	0.067 (−0.048; 0.182)	0.06 (−0.038; 0.158)	0.063 (−0.041; 0.168)	0.072 -0.038; 0.182)	0.066 (−0.059; 0.191)	0.073 (−0.060; 0.207)	**0.535 (0.425; 0.645)**
TTO	20	8.6	**0.151 (0.098; 0.203)**	**0.209 (0.086; 0.331)**	**0.180 (0.097;0.264)**	**0.191 (0.096; 0.285)**	**0.199 (0.091; 0.307)**	**0.207 (0.080; 0.334)**	**0.213 (0.079; 0.348)**	**0.555 (0.377; 0.733)**
15D	2	0.9	0.017 (−0.059; 0.094)	0.069 (−0.072; 0.211)	0.040 (−0.064; 0.144)	0.065 (−0.063; 0.193)	0.064 (−0.054; 0.182)	0.067 (−0.072; 0.205)	0.076 (−0.076; 0.227)	**0.493 (0.335; 0.651)**
SF6D	7	3	**0.171 (0.11; 0.231)**	**0.214 (0.077; 0.350)**	**0.189 (0.095; 0.283)**	**0.199 (0.093; 0.306)**	**0.201 (0.095; 0.307)**	**0.201 (0.078; 0.325)**	**0.217 (0.074; 0.360)**	**0.613 (0.455; 0.771)**
HUI	31	13.3	−0.052 (−0.127; 0.022)	−0.042 (−0.178; 0.093)	−0.055 (−0.159; 0.049)	−0.044 (−0.159; 0.072)	−0.041 (−0.153; 0.071)	−0.052 (−0.180; 0.076)	−0.04 (−0.187; 0.106)	**0.319 (0.173; 0.465)**
EQ5D	73	31.2	**0.114 (0.048; 0.179)**	0.124 (−0.006; 0.254)	**0.113 (0.016; 0.210)**	**0.124 (0.015; 0.234)**	**0.125 (0.021; 0.229)**	0.116 (−0.005; 0.237)	0.127 (−0.015; 0.268)	**0.541 (0.407; 0.676)**
**Length of ART**										
Length of ART _1										**−0.148 (−0.274; −0.021)**
Length of ART _2										**0.397 (0.034; 0.760)**
**Study design**										
Cross-sectional	123	52.6								
Longitudinal	111	47.4						0.033 (−0.027; 0.092)		
**Disease stages**										
Asymptomatic	22	9.4								
Symptomatic	41	17.5	−0.017 (−0.094; 0.059)				−0.025 (−0.084; 0.034)			
AIDS	67	28.6	**−0.173 (−0.261; −0.086)**				**−0.176 (−0.278; −0.075)**			
Mixed group	104	44.4	**−0.061 (−0.120; −0.002)**				−0.057 (−0.124; 0.009)			
**Treatment**										
Non ART	63	26.9								
ART	150	64.1	**−0.076 (−0.150; −0.002)**			−0.067 (−0.170; 0.036)				
Combined	21	9	−0.015 (−0.074; 0.045)			0.025 (−0.016; 0.066)				
**Setting**										
Developed	182	77.8								
Developing	52	22.2	**−0.082 (−0.163; −0.001)**		−0.094 (−0.216; 0.029)					**−0.087 (−0.187; 0.012)**
**Year**										
2000/4	38	16.2								
2005/9	149	63.7	−0.023 (−0.074; 0.029)	−0.017 (−0.094; 0.059)						**0.196 (0.067; 0.325)**
2010/3	47	20.1	0.039 (−0.036; 0.115)	0.014 (−0.036; 0.064)						**0.248 (0.064; 0.431)**

Figures in bold: p-values<0.05.

Type of instrument used was a significant predictor of health utility estimates. Adjusting for study characteristics, the SF-6D and the HUI yielded the highest and lowest scores, respectively. We found large, statistically significant differences between utility elicited by SF-6D (0.171), EQ-5D (0.114), and TTO (0.151) and the reference measure, SG. Meanwhile, VAS and HUI provided utility estimates that were not significantly different than SG.

Compared to asymptomatic HIV patients, symptomatic and AIDS patients reported a decrease in utility score of 0.025 (p = 0.40) and 0.176 (p = 0.001), respectively, when adjusting for method of assessment, 0.017 (p = 0.65) and 0.173 (p < 0.001), respectively, when adjusting for all study characteristics.

Health utility of HIV/AIDS patients in developing countries was 0.082 lower than those who lived in developed countries. We did not find significant differences in utility estimates across different years of publication.

### Longitudinal changes in health utility of HIV/AIDS patients

**Figure 2:**
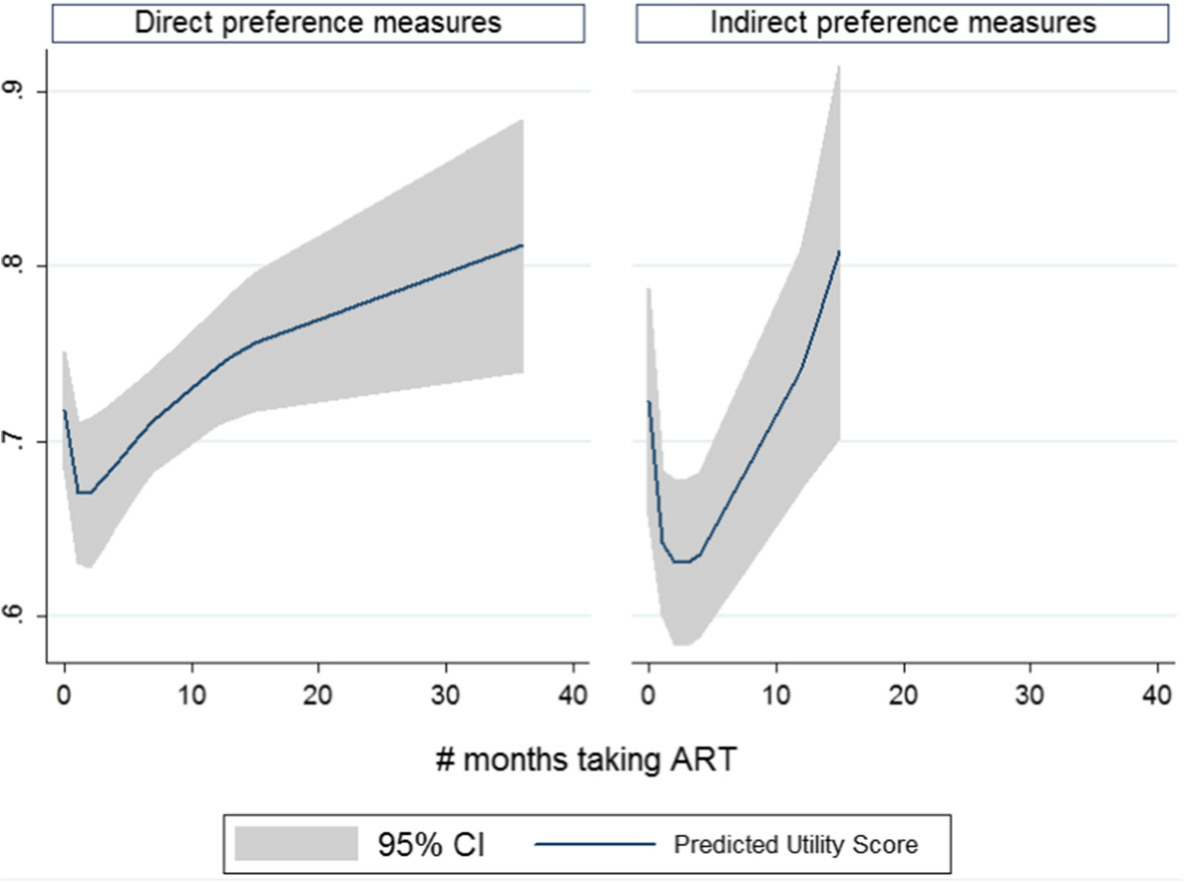
**Predicted utility score and 95% confidence interval by types of measures based on the best fitting fractional polynomial model.**

**Figure 3:**
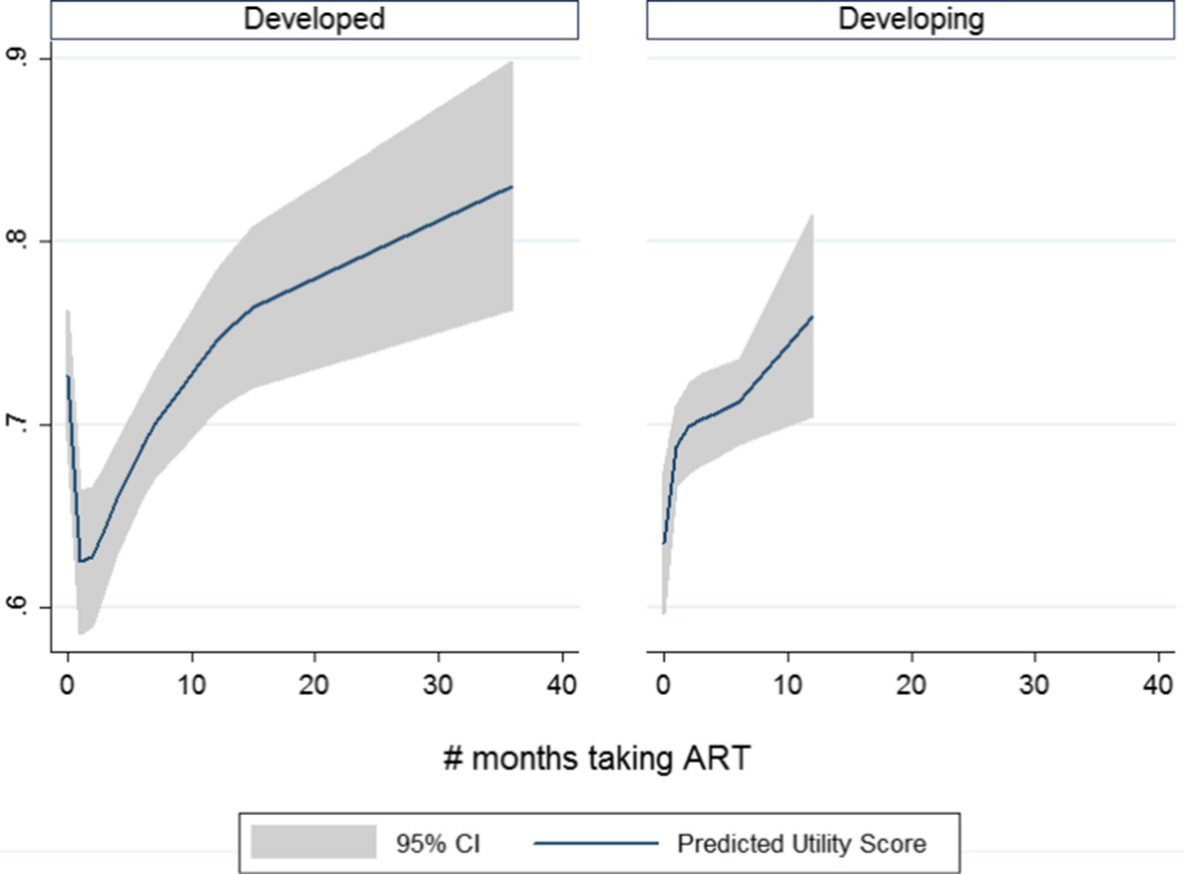
**Predicted utility score and 95% confidence interval by study settings based on the best fitting fractional polynomial model.**

We used a multivariate fractional polynomial model of the 99 utility measurements from the 14 selected longitudinal studies to analyse changes in health utility over time (see Table 5-Model 8, Figures 2 and 3). The model’s coefficients show that the duration of ART was a significant predictor of the changes in health utility scores of HIV/AIDS patients, after adjusting for study characteristics. Health utility of HIV/AIDS patients significantly decreased in the first 3 months of treatment, and rapidly increased afterwards (Figure 2). The magnitude of change was also affected by duration of ART, as well as by the methods of assessment. Direct preference-based measures resulted in greater changes in utility scores than indirect preference-based measures during the first year of treatment. Starting from the second year, though, the magnitude of change in health utility measured by indirect-preference instruments was larger than direct-preference ones. While this trend was typical for studies conducted in developed countries, it was slightly different in developing countries. In such countries as South Africa, Brazil, Thailand, Uganda, and Vietnam, patients’ health utility markedly increased right after the initiation of ART, and then changed only slightly during the first 6 months of treatment, before increasing rapidly again afterwards (Figure 3).

## Discussion

By systematically reviewing studies of health utility among HIV/AIDS patients, we provide an accumulation of psychometric evidence of the preference-based HRQL instruments applied in this patient group. Moreover, we compared the performance and utility estimates by various instruments, as well as modelled the changes in health utility over the course of HIV/AIDS treatment. Prior to this work, Tengs and Lin did a meta-analysis of health utility estimates from studies published from 1985–2000 [16]. In this study, we found similar findings that disease stage is an important predictor of health utility. Also, different HRQL instruments might yield clinically important differences in health utility scores. Moreover, findings of this study provide most-updated evidence of preference-based HRQL assessments among patients with HIV/AIDS during 2000–2013. This is the period when HIV/AIDS treatment services have been rapidly scaled up in developing countries. We extend previous work by analyzing the changes in health utility of patients over the course of ART. Especially, we revealed that different types of instruments had different levels of responsiveness over the early and stable periods of ART.

When analyzing the performance of the different instruments, we found that the Time Tradeoff (TTO) instrument, SF-6D, and EQ-5D yielded higher utility scores than the reference Standard Gamble (SG) instrument, while the Visual Analogue Scale (VAS), HUI, and 15D showed no statistically significant difference in measurement than the SG. This is in contrast to various other studies, in which the use of the SG method generally yields the highest utility score among direct-preference instruments [8,72]. Generally, it is believed that SG yields higher health utilities, because it asks patients to make a gamble between a chance of good health and a chance of death, and most people are reluctant to accept a large risk of death to avoid an adverse health state [72,73]. There has been very little research about the effect of context on SG and TTO instruments, and yet our results indicate that these instruments may perform differently in HIV/AIDS populations [74]. Indeed, one of the papers included in this review showed that SG was an unreliable measurement of healthy utility in HIV/AIDS patients (0.41) and that TTO and VAS were much more reliable (0.71-0.83) [17]. This low reliability may help explain why SG yielded lower utility scores, contrary to what was expected.

Given that HIV is a chronic disease that changes over time, it is essential that HRQL measures are responsive to clinically significant changes the patient experiences. Most of the indirect measures included in this study were responsive to opportunistic infections, clinical AIDS-defining events, adverse events, or global rating of change, and the direct preference-based measures were able to discriminate subjects by disease severity. When analysing the performance of measures throughout the duration of ART, we found that during the first year of treatment, direct preference-based measures resulted in greater changes in utility scores than indirect preference-based measures, but starting from the second year, this trend reversed and indirect preference-based measures resulted in great changes than direct preference measures. This may be due to the fact that direct preference-based measures may reflect the change in subjects’ perception of their health status rather than a true change in health status [74,75]. Therefore, change in utility, in short term, might be influenced by the hope of HIV patients getting treated [19]. Similarly, in the long-term, patients may become acclimated to their health status, and thus become more likely to report a high health status [3].

These results provide some evidence to inform the selection of preference-based HRQL instrument for measuring health utility of HIV/AIDS patients. First, given the variation of health utility scores based on type of instrument used, and given the difference in direct versus indirect preference measures over time, researchers should combine both direct and indirect preference-based instruments. This could be done by incorporating VAS into other health status profiles. Our review also highlights the limitations of using generic measures of HRQL in HIV/AIDS patients. Our meta regression showed steady growth of health utility scores over the stable period of ART, however many side-effects of ART, including lipodystrophy, sleep disturbance, and sexual functioning, have negative effects on patients’ quality of life and should be reflected in HRQL measures [12,13,76,77]. Additionally, some tools, such as EQ-5D-3L, showed a high ceiling effect, for example, which may affect their responsiveness in monitoring the outcomes of ART. These findings suggest that it may be beneficial to develop more HIV-specific questionnaires that are sensitive to ART. Finally, we found that these measures performed differently in developed and developing countries. Patients in developed countries had higher health utility scores than patients from the developing world. Additionally, patients in developed countries demonstrated an initial decrease in HRQL upon initiation of ART, followed by a large and steady increase, whereas patients in developed countries experienced a large initial increase, following by little change in the first 6 months, and large steady increase again after that. Since developing countries have a greater burden of HIV/AIDS, it may be of great interest for further studies to examine the determinants of HRQL of patients with HIV/AIDS specifically in this setting.

Finally, our use of pooled health utility estimates to determine the changes in HRQL during treatment has significant implication for economic and clinical evaluation of HIV/AIDS care and treatment interventions. In particular, the rapid reduction in health utility during the first 3 months of ART highlights the importance of intensive support for patients after ART initiation to relieve both physical and psychological burden experienced by these patients.

The strengths of this meta-analysis include a systematic approach in synthesizing evidence from the literature. In addition, we applied multivariate fractional polynomial models to select the best fitting model for changes in health utility and length of ART. However, there are some limitations to be acknowledged. First, aggregated data in some studies limited the estimate ability of the model. Second, the length of ART was inconsistent between different patient groups and health utility measures. Third, the frequency of application of some instruments, such as 15D and HUI2, was very small, which resulted in imbalanced models. Finally, since the selected measures are generic instruments, we were not able to identify a set of common measures, including HIV-specific items, to be used for comparing across studies.

The pooled estimates of health utilities and trends throughout the course of ART provided in this study provide valuable information about the effect of ART on HIV/AIDS patients health related quality of life, which in turn can support developing economic models for evaluating the cost-effectiveness of HIV/AIDS treatment strategies. Researchers can use estimated utility scores by this study for quantifying time-dependent health outcomes of interventions in their cost-effectiveness models. In addition, significant reductions in health utility during the first six month on ART suggest that additional care and support and intensive monitoring should be incorporated in clinical practice. Finally this study provides a basis for the selection of preference-based HRQL instruments for future research in HIV population.

## Conclusion

The study provides an accumulation of evidence on measurement properties of health utility estimates that can help inform the selection of instruments for future studies. The pooled estimates of health utilities and their trends are useful in economic evaluation and policy modelling of HIV/AIDS treatment strategies.

## Additional files

Additional file 1:
**Syntax for data analysis.**
Additional file 2:
**Pooled health utility data for STATA software.**
