# Supplementary material for: Longitudinal and cross sectional assessments of health utility in adults with HIV/AIDS: a systematic review and meta-analysis
Source: BMC Health Serv Res. 2015 Jan 22;15:7. doi: 10.1186/s12913-014-0640-z (PMC4307193; doi:10.1186/s12913-014-0640-z)
Supplement: Additional file 1: — Syntax for data analysis. [file 12913_2014_640_MOESM1_ESM.docx]

**ANNEX 1: SYNTAX**

1. **For all measurements (n=218)**

**Model 1:**

xtmixed u i.tool2 i.stage2 i.art2 i.year2 i.developing || id: tool2 design, var vce(robust) nocons cov(uns)

**Model 2:**

xtmixed u i.tool2 i.year2 || id: tool2 design, var vce(robust) nocons cov(uns)

**Model 3:**

xtmixed u i.tool2 i.developing || id: tool2 design, var vce(robust) nocons cov(uns)

**Model 4:**

xtmixed u i.tool2 i.art2 || id: tool2 design, var vce(robust) nocons cov(uns)

**Model 5:**

xtmixed u i.tool2 i.stage2 || id: tool2 design, var vce(robust) nocons cov(uns)

**Model 6:**

xtmixed u i.tool2 i.design || id: tool2 design, var vce(robust) nocons cov(uns)

**Model 7:**

xtmixed u i.tool2 || id: tool2 design, var vce(robust) nocons cov(uns)

1. **For measurements within longitudinal studies (n=99)**

**Model 8:**

xi: mfp: glm u i.tool2 lengart i.year2 i.developing if design==1, vce(robust) nocons
